# Resin-dentin bond stability of etch-and-rinse adhesive systems with different concentrations of MMP inhibitor GM1489

**DOI:** 10.1590/1678-7757-2019-0499

**Published:** 2020-04-27

**Authors:** Maria Elisa da Silva Nunes Gomes MIRANDA, Eduardo Moreira da SILVA, Mariana Flôres de OLIVEIRA, Fabiana Souza SIMMER, Glauco Botelho dos SANTOS, Cristiane Mariote AMARAL

**Affiliations:** 1 Universidade Federal Fluminense Departamento de Odontotécnica LABIOM-R NiteroiRio de Janeiro Brasil Universidade Federal Fluminense, Departamento de Odontotécnica, LABIOM-R, Niteroi, Rio de Janeiro, Brasil.

**Keywords:** Dentin-bonding agents, Matrix metalloproteinase inhibitors, Dental Restoration

## Abstract

Enzymatic degradation of the hybrid layer can be accelerated by the activation of dentin metalloproteinases (MMP) during the bonding procedure. MMP inhibitors may be used to contain this process.

**Objective:**

To evaluate the degree of conversion (DC%), dentin bond strength (µTBS) (immediate and after 1 year of storage in water), and nanoleakage of an experimental (EXP) and a commercial (SB) adhesive system, containing different concentrations of the MMP inhibitor GM1489: 0, 1 µM, 5 µM and 10 µM.

**Methodology:**

DC% was evaluated by FT-IR spectroscopy. Dentin bond strength was evaluated by µTBS test. Half of beams were submitted to the µTBS test after 24 h and the other half, after storage for 1 year. From each tooth and storage time, 2 beams were reserved for nanoleakage testing. Data were analyzed using ANOVA and Tukey’s test to compare means (α=0.05).

**Results:**

All adhesive systems maintained the µTBS after 1 year of storage. Groups with higher concentrations of inhibitor (5 µM and 10 µM) showed higher µTBS values than groups without inhibitor or with 1 µM. The nanoleakage values of all groups showed no increase after 1 year of storage and values were similar for SB and EXP groups, in both storage periods. The inhibitor did not affect the DC% of the EXP groups, but the SB5 and SB10 groups showed higher DC% values than those of SB0 and SB1.

**Conclusions:**

The incorporation of GM1489 in the adhesive systems had no detrimental effect on DC%. The concentrations of 5 µM GM1489 for SB and 5 µM or 10 µM for EXP provided higher μTBS than groups without GM1489, in the evaluation after 1 year of storage; whereas the concentration of inhibitor did not affect adhesive systems nanoleakage.

## Introduction

Advances in adhesive Dentistry have led to increased immediate bond strength of resin composites to dentin, but the resin-dentin bonds are not as durable as resin-enamel bonds. Degradation of the hybrid layer may occur at various levels and stages,^1 , 2^ as well as degradation of the unprotected collagen fibrils^2^ because of its incomplete permeation by dentin adhesive or by elution of unreacted monomers and oligomers. These unprotected fibrils are prone to proteolytic degradation by metalloproteinases (MMP),^3^ which are a family of endogenous proteolytic enzymes capable of degrading all components of the extracellular matrix.^4^ The MMP 1, 2, 3, 8, 9 and 20 have been identified in dentin and saliva. However, the collagenase MMP 8 and gelatinases MMP 2 and 9 have been identified as being key enzymes in the process of degradation of the collagen matrix in dentin.^5^ The MMP present in dentin are inactive and can be activated by changes in pH during caries lesion progression^5^ or in adhesive protocol.^6 , 7^ Other proteolytic enzymes that act on dentin are the cysteines-cathepsins, which seem to act on caries progression and degradation of the hybrid layer.^8 , 9^ Possibly, cysteines-cathepsins and MMP act synergistically in an enzymatic cascade of degradation of the collagen matrix.^8^

Some studies have shown that MMP inhibitors such as Chlorhexidine, Batimastat, Galardin, and EDTA can improve the integrity and stability of the resin-dentin bond when used as a dentin pretreatment, before resin infiltration.^4 , 10 , 11^ However, the results regarding the use of MMP inhibitors in dentin bonding are still controversial and in few studies, the inhibitor was incorporated into the adhesive system.^12 - 14^ Chlorhexidine is the most frequently investigated MMP inhibitor, and it is capable of reducing dentin-resin degradation when added to experimental^14 , 15^ and commercial^16 - 18^ adhesive systems, in addition to decreasing the gelatinolytic activity of MMP.^18 , 19^ However, this inhibitor is susceptible to leaching in a short period of time, which interrupts the inactivation of the MMP, promoting degradation of the exposed collagen fibrils at the adhesive interface and decreasing the resin-dentin bond strength.^20 , 21^ Other MMP inhibitors such as Batimastat and Galardin have been incorporated into adhesive systems.^13 , 14 , 22^ In previous study, Galardin and Batimastat were able to inhibit the MMP of dentin, but they were not capable of maintaining the µTBS after three months of storage.^13^ On the other hand, another study showed that Batimastat, Chlorhexidine and GM1489, in experimental adhesive, were capable of maintaining the µTBS after 12 months of storage, different from Control and Galardin;^14^ Batimastat and GM1489 also maintained resin-dentin bond stability after 12 months for superficial and deep dentin, different from Chlorhexidine and the control group.^22^

Another broad-spectrum synthetic MMP inhibitor, GM1489 (C_27_H_33_N_3_O_4_: N-[(2R)-2-(Carboxymethyl)-4-methylpentanoyl]-L-tryptophan-(S)-methyl-benzylamide), which has inhibitory action on MMP 1, 2, 3, 8 and 9 have been used in the medical field.^23 , 24^ It has shown promising results regarding resin-dentin bonding stability when added to experimental and commercial total etch adhesive system.^14 , 22^ However, there is still little information available about this inhibitor.

Therefore, this study aims to evaluate the influence of different GM1489 concentrations (0, 1 µM, 5 µM and 10 µM) on the stability of bond strength to dentin, nanoleakage and degree of conversion of commercial and experimental adhesive systems. The hypotheses tested were: 1) higher concentrations of GM1489 could preserve the dentin bond strength after 1 year of storage, 2) higher concentrations of GM1489 could reduce the nanoleakage at the adhesive interface, and 3) higher concentrations of GM1489 could not affect the degree of conversion of adhesive systems.

## Methodology

### Synthesis of the experimental adhesive systems

The experimental adhesive system (EXP) was formulated as in previous study,^14^ using the following monomers (wt.%): HEMA (25%), 4-META (30%), TEGDMA (25%) (Essthec, Inc. Essington, PA, USA). Acetone (15%) and water (4%) were used as solvents and Camphorquinone (0.5%) and EDMAB (0.5%) (Ethyl 4-(dimethylamino)benzoate – Aldrich Chemical Company, Inc., Milwaukee, WI, USA) were incorporated as photosensitizer and reducing agents, respectively. The components of the adhesive were weighed using an analytical balance (AUW 220D, Shimadzu, Tokyo, Japan), mixed and homogenized in a dual centrifuge (150.1 FVZ SpeedMixer DAC, FlackTek Inc., Herrliberg, Germany) at 1300 rpm for 2 minutes.

**Figure 1:**
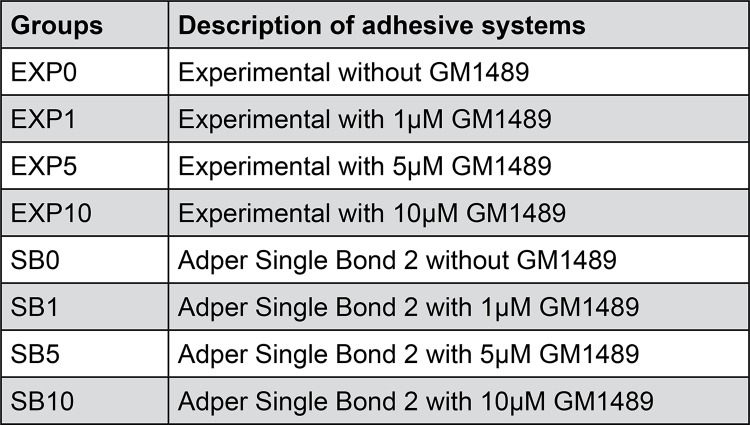
Experimental groups and adhesive systems used in this study

The MMP inhibitor GM1489 (EMD Chemicals, Inc. San Diego, CA, USA) was incorporated in this formulation in different concentrations 0, 1 µM, 5 µM and 10 µM, obtaining four different experimental adhesives. The adhesive system Adper Single Bond 2 (SB) (3M ESPE, Sumaré, SP, Brazil) was used as a commercial reference and also received different concentration of GM1489. After incorporation of GM1489 in each adhesive system, they were homogenized at 2400 rpm for 2 min.^14^Figure 1 shows the tested groups.

### Microtensile bond strength (μTBS) measurement

A total of 48 extracted, caries free, human third molars (Research Ethics Committee Approval HUAP CAAE 47695315.2.0000.5243) were disinfected in 0.5% chloramine T solution for 7 days, stored in distilled water and used within six months after extraction. The *μ* TBS measurement was performed according to da Silva, et al.^14^ (2015). The occlusal dentin of the teeth was exposed using a cut machine (IsoMet 1000, Buëhler, Lake Bluff, IL, USA) and the peripheral enamel was removed using a diamond bur (#4138, KG Sorensen, Cotia, SP, Brazil). The smear layer of dentin was standardized with 600-grit SiC papers (Arotec, Cotia, SP, Brazil) in politriz (DPU 10, Struers, Denmark) for 1 minute. After preparation of the dentin surfaces, the teeth were divided into eight groups (n=6) according to the adhesive system tested ( Figure 1 ).

Dentin surfaces were etched with 37% phosphoric acid for 15 seconds (Condac37, FGM, Joinville, SC, Brazil), rinsed with distilled water for 30 seconds and blot dried with absorbent paper. Two consecutive layers of each adhesive system were applied on active mode, followed by gentle air stream for 5 seconds and light curing for 20 seconds with an irradiance of 650 mW/cm^2^ (DEMI, Kerr Corporation, Middleton, WI, USA). Five increments of 1 mm thick resin composite (Filtek Z250, 3M Espe, St Paul, MN, USA) were horizontally added to the bonded surfaces and individually light cured for 40 seconds with an irradiance of 650 mW/cm^2^.^14^

After storage in distilled water at 37°C for 24 h, the teeth were longitudinally sectioned in both mesio-distal and buccal-lingual directions, across the bonded interfaces (IsoMet 1000, Buëhler, Lake Bluff, IL, USA) to obtain beams with a cross-sectional area of approximately 1 mm^2^.^14^ Each tooth provided 15 to 23 beams. Two beams of each tooth were preserved for the nanoleakage test (immediate and 1 year). The remaining beams were divided into two subgroups according to the time of storage in distilled water at 37 °C: immediate and ١ year.

After each period of storage, the beams had their adhesive interfaces cross-sectional area measured with a digital caliper (MPI/E-101, Mytutoyo; Tokyo, Japan) and were individually fixed to a microtensile device (ODMT03d, Odeme Biothecnology, Joaçaba, SC, Brazil) using cyanoacrylate glue (Superbonder Gel, 3M, São Paulo, SP, Brazil) and loaded under tension using a universal testing machine (EMIC DL 2000, São José dos Pinhais, SP, Brazil) at a crosshead speed of 0.5 mm/min until failure occurred. The μTBS (MPa) was obtained by dividing the load at failure (N) by the cross-sectional area of each tested beam (mm^2^). The fractured surfaces were evaluated under stereomicroscope at 40x magnification (SZ40, Olympus, Tokyo, Japan) and failure modes were classified as: adhesive (failures at the adhesive interface), cohesive (failures occurring in dentin or in resin composite), or mixed (mixture of adhesive and cohesive failure within the same fractured surface).^14^ Additionally, representative fractured beams exhibiting different failure modes and with μTBS value close to the mean of each group were observed using Laser Confocal microscope (Lext OLS4001, Olympus, Center Valley, PA, USA) operating on scanning mode XYZ fast scan, at 50x magnification (lens MPLAPONLEXT 50).

### Nanoleakage

After storing (immediate or 1 year), two beams of each tooth were prepared for the nanoleakage test as previously described.^22^ The beams received two layers of nail varnish up to 1 mm from the bonding interface on both sides and were individually immersed in 50 wt% ammoniacal silver nitrate solution (pH=7.0) in a dark environment for 24 hours. Each beam was thoroughly rinsed in running water and then immersed in a photo-developing solution (Kodak, Rochester, New York, NY, USA) under fluorescent light for 8 hours, to reduce silver ions into metallic silver grains at the bonding interface. Afterwards, the surfaces were wet polished with 600-grit, 1200-grit and 4000-grit silicon carbide paper, ultrasonically cleaned in water for 10 minutes (Ultrassom 750 USC – Quimis, Rio de Janeiro, RJ, Brazil) and dried for 48 hours in a desiccator with blue silica gel at 37°C.

The resin/dentin interface were observed using scanning electron microscopy (SEM) (Phenom ProX, Phenom-World BV, Eindhoven, Netherlands), at an accelerating voltage of 15 kV, backscattered mode, and using a charge reduction sample holder (low vacuum environment). Three images were registered for each beam: two from both ends (right and left sides) and one central, with a magnification of 2000x.^22^ In these images, the amount of silver nitrate uptake in the hybrid layer was registered as a percentage of the total area observed, using an Energy-dispersive X-ray spectroscopy detector (Phenom ProX, Phenom-World BV, Eindhoven, Netherlands). The percentage of Ag of each image was recorded. The mean percentage of Ag of the three images (right, left and central) was estimated and considered as the experimental unit (n=6).

### Degree of conversion (DC%)

Increments of each adhesive system were inserted into a Teflon mold (0.785 mm^3^) positioned onto a crystal, using attenuated total reflection mode of the FT-IR spectrometer (Alpha-P/Platinum ATR Module, Bruker Optics GmbH, Ettlingen, Germany) and the spectra between 1600 and 1800 cm^-1^ were recorded with the spectrometer operating with 40 scans, at a resolution of 4 cm^-1^.^14^

Afterwards, the increments were light-cured for 20 seconds with an irradiance of 650 mW/cm^2^ (DEMI, Kerr Corporation, Middleton, WI, USA) and the spectra were recorded exactly as it was performed for the unpolymerized increments. Each adhesive system was evaluated in triplicate (n=3). The DC% was estimated from the ratio between the integrated area of absorption bands of the aliphatic C=C bond (1638 cm^-1^) to that of the C=O bond (1720 cm^-1^), used as an internal standard, which were obtained from the polymerized and unpolymerized increments,^14^ using the following equation:

$$\mathrm{DC}\%=100x[1–(R_{\mathrm{polymerized}}/R_{\mathrm{unpolymerized}})],$$

where R = integrated area at 1638 cm^-1^ / integrated area at 1720 cm^-1^

### Statistical analysis

The obtained data were analyzed using Statgraphics Centurion XVI software (STATPOINT Technologies Inc, Warrenton, VA, USA). Initially, the normal distribution of errors and homogeneity of data variances were checked using Shapiro-Wilk’s and Levene’s test, respectively.^14 , 22^ Based on these preliminary analyses, the DC% was evaluated by two-way ANOVA (GM1489 concentration and adhesive system) and Tukey’s HSD *post hoc* test. Nanoleakage and μTBS data were analyzed using three-way ANOVA (GM1489 concentration, adhesive system and storage time) and Tukey’s HSD *post hoc* test for multiple comparisons. The analyses were performed at a significance level of 5%.

## Results

The µTBS results are shown in Table 1 . Three-way ANOVA test showed statistical significance for the independent factors: adhesive (p=0.0499) and inhibitor concentration (p=0.000). Additionally, the interactions adhesive vs. inhibitor concentration (p=0.0000), and adhesive vs. inhibitor concentration vs. time (p=0.0451) were significant. The independent factor time (p=0.2292) and the interactions adhesive vs. time (p=0.1672) and inhibitor concentration vs. time (p=0.0984) were not significant. In the immediate time interval, µTBS values of EXP10 were significantly higher than those of EXP0 and EXP1, but without difference from EXP5. EXP0, EXP1 and EXP5 presented similar µTBS. For SB the immediate µTBS value was similar for all groups (SB0, SB1, SB5 and SB10).

**Table 1:** Mean and standard deviation values of µTBS (MPa) after each period of storage in distilled water

Adhesive System	Period of test	
	Immediate	1 year storage
EXP0	27.43 (5.4)^Ba^	21.21 (4.8)^Ca^
EXP1	21.42 (4.2)^Ba^	22.21 (2.6)^BCa^
EXP5	31.02 (7.2)^ABa^	29.43 (8.2)^ABa^
EXP10	43.36 (4.1)^Aa^	36.19 (3.3)^ABa^
SB0	31.52 (9.9)^Aa^	26.25 (7.8)^BCa^
SB1	35.02 (4.5)^Aa^	27.95 (7.0)^ABa^
SB5	30.85 (5.9)^Aa^	40.15 (6.0)^Aa^
SB10	27.09 (8.3)^ABa^	31.12 (7.4)^ABa^

Means followed by different letters (uppercase – column, lowercase - row) are statistically different (Tukey´s HSD test, a = 0.05)

**Figure 2:**
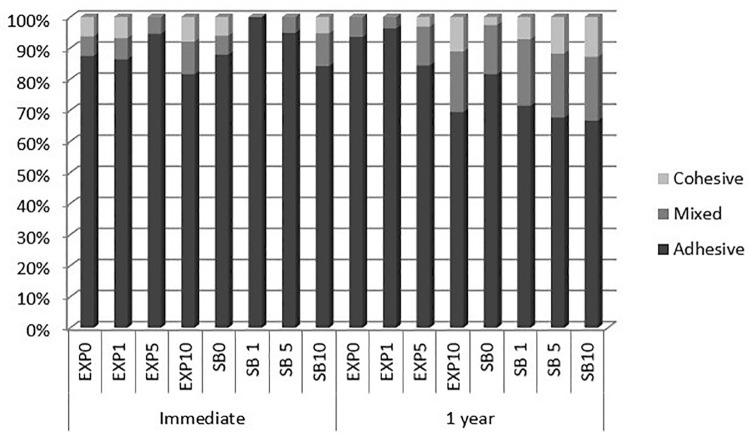
Failure mode (%) of each group after each period of storage in distilled water

**Figure 3:**
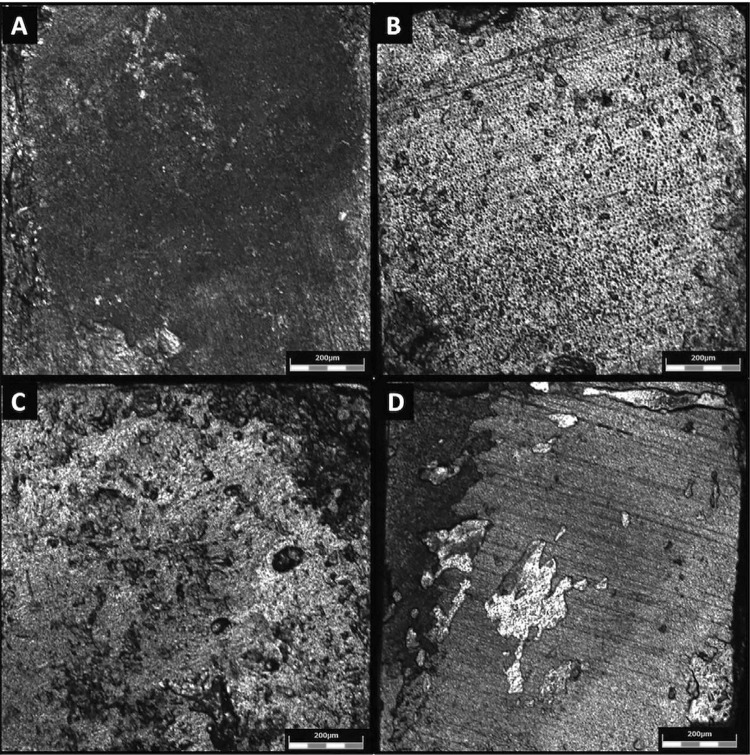
Representative images of the failure modes of beams after µTBS measurement. A: Adhesive failure of group EXP5 in immediate time; B: Adhesive failure of group SB1 in immediate time; C: Mixed failure of group EXP10 after 1 year of storage; D: Mixed failure of group SB1 after 1 year of storage

After 1 year of storage, only SB5 presented significantly higher µTBS values than SB0. SB1, SB10 and SB0 showed similar µTBS values. For EXP, the groups EXP5 and EXP10 showed significantly higher µTBS values than EXP0. However, EXP1 showed µTBS values similar to EXP0, EXP5 and EXP10. In all groups the µTBS value after 1 year of storage was similar to the µTBS value found in the immediate time interval. Figure 2 presents the failure mode analysis, which showed predominantly adhesive failures in all groups. Some images of failure patterns (adhesive and mixed) are shown in Figure 3 .

**Figure 4:**
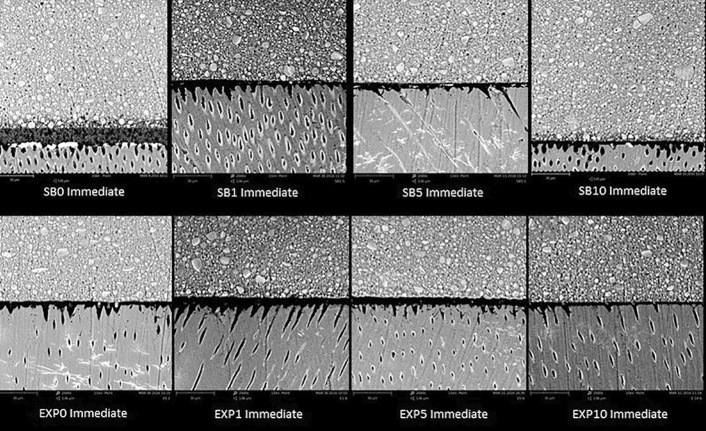
Representative Immediate back-scattering SEM images of the resin-dentin interfaces bonded with SB and EXP in all concentrations of the inhibitor

**Figure 5:**
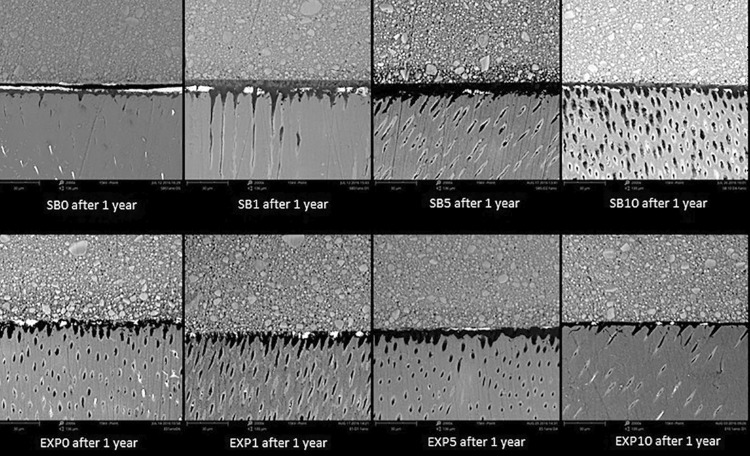
Representative back-scattering SEM images after 1 year of storage of the resin-dentin interfaces bonded with SB and EXP in all concentrations of the inhibitor

The nanoleakage results are summarized in Table 2 . Three-way ANOVA showed statistical significance for the independent factors: adhesive (p=0.0001), inhibitor concentration (p=0.009) and time (p=0.000). The interaction adhesive vs. inhibitor concentration was also significant (p=0.0083), while the other interactions were not significant (adhesive vs. time p=0.2352; inhibitor concentration vs. time p=0.2129; adhesive vs. time vs. inhibitor concentration p=0.8122). In the immediate time interval and after 1 year of storage, differences among the nanoleakage of groups were not observed for EXP (EXP0, EXP1, EXP5 and EXP10) and for SB (SB0, SB1, SB5 and SB10). The nanoleakage of all groups did not increase after 1 year of storage, except for EXP0. Representative SEM images of nanoleakage of adhesive systems are shown in Figures 4 and 5 .

**Table 2:** Mean and standard deviation values of nanoleakage (Ag percentage) after each period of storage in distilled water

Adhesive System	Period of test	
	Immediate	1 year storage
EXP0	0.59% (0.21%)^ABa^	1.20% (0.18%)^ABb^
EXP1	1.04% (0.44%)^ABa^	1.17 (0.10%)^ABa^
EXP5	0.67% (0.16%)^ABa^	1.09 (0.24%)^Aa^
EXP10	0.48% (0.11%)^Aa^	0.81 (0.13%)^Aa^
SB0	1.28% (0.68%)^Ba^	2.04 (0.81%)^Ba^
SB1	1.09% (0.31%)^ABa^	1.48 (0.15%)^ABa^
SB5	0.72% (0.16%)^ABa^	1.31 (0.53%)^ABa^
SB10	0.66%(0.18%)^ABa^	1.37 (0.21%)^ABa^

Means followed by different letters (uppercase – column, lowercase - row) are statistically different (Tukey´s HSD test, a = 0.05)

**Figure 6:**
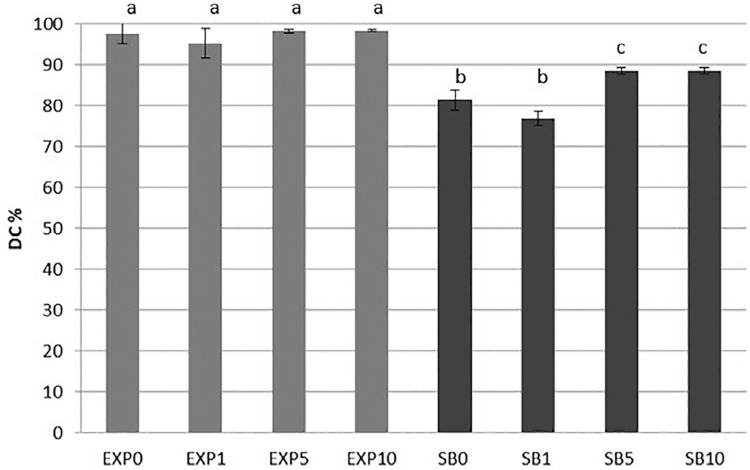
Mean DC% for adhesive systems. Different letters indicated statistically significant difference by Tukey’s test (α=0.05)

The results of DC% are presented in Figure 6 . Two-way ANOVA detected a statistical significance for the independent factors adhesive (p=0.0000) and inhibitor concentration (p=0.0000) as well as for the interaction between these two factors (p=0.0022). The incorporation of the inhibitor GM 1489 did not affect the DC% of experimental adhesive systems. Whereas, the addition of 5 µM or 10 µM of GM١٤٨٩ to SB showed significant increase in DC%.

## Discussion

In this study, a simple formulation of an adhesive blend was used based on that by da Silva, et al.^14^ (2015). This occurred because the commercial adhesive systems available did not detail the exact amount of each component present in their formulations, so that undesirable and unknown effects could have endanger the discussion of results obtained here. On the contrary, a known adhesive formulation allowed a better discussion about the interactions that happened between the different concentrations of MMP inhibitor GM1489 with the adhesive formulation. Different from previous studies that have used different MMP inhibitors as pretreatment for dentin substrate,^4 , 10 , 11^ in this study, GM1489 was directly added to the experimental and commercial adhesive systems in an endeavor to eliminate one step in the restorative process.

For this purpose, a 4-META-based experimental adhesive system was formulated. The 4-META is a functional monomer that has been used as an adhesion promoter and as demineralizing monomer.^25^ By reacting with water, 4-META monomer is hydrolyzed to 4-MET, which is able to establish an ionic bonds with Ca^2^ in hydroxyapatite.^26 - 28^ This chemical interaction during the formation of the hybrid layer may improve the durability of the adhesive restorations.^1^ GM1489 was chosen because it is a MMP inhibitor of broad spectrum that has not been extensively studied in Dentistry. Also, in a previous study, this inhibitor has maintained the bond of an experimental adhesive system to dentin after 12 months of storage when used in the 5 µM concentration.^14^ GM 1489 is an acetohydroxamic acid that contains the critical metal ligand group and a complex heterocyclic structure, which could favor its chelation potential.^14 , 22^ Similarly to Galardin, the GM1489 can bind to the active site of MMP, chelating the zinc ion that is located in the catalytic domain of MMP.^19^ GM1489 presents the following *in vitro* inhibitory constants (K_i_): MMP 1=0.2 nM, MMP 2=500 nM, MMP 3=20 µM, MMP 8=100 nM, and MMP 9=100 nM. Therefore, it was reasonable to claim that lower concentrations of GM1489 could inhibit the activity of MMP 2, 8 and 9, thereby preventing the degradation of hybrid layer over time. This was the reason for testing the 1 µM concentration in this study. The concentration of 5µM was based on the results by da Silva, et al.^14^ (2015) and the higher 10 µM concentration as a function of the inhibitory constant for MMP3. The commercially available adhesive system (SB) was used to evaluate whether a different adhesive composition would influence the effectiveness of GM1489 on dentin bond stability.

Indeed, when 10 µM GM1489 was used with the experimental adhesive system, the µTBS values were the highest in both time intervals of evaluation, but without significant difference from 5 µM. The initial µTBS was also increased by use of 5 µM and 10 µM of GM1489 for the experimental adhesive system, but the same did not occur with SB. On the other hand, for SB the greatest µTBS after 1 year of storage occurred when 5 µM GM1489 was used, but without significant difference from 1 µM and 10 µM. Thus, the second research hypothesis that higher concentrations of GM1489 would preserve the bond strength to dentin after 1 year of storage was partially accepted. These results showed that the composition of adhesive systems could influence the optimal concentration of GM1489 required to improve the dentin bond strength. Moreover, the µTBS did not decrease after 1 year of storage, for all groups. This may indicate that longer time of storage may be required to highlight the effect of GM1489 on resin-dentin bond preservation, although its use has caused increase of dentin µTBS.

In the immediate µTBS test, adhesive failures were predominant, which could indicate the reliability of the test and demonstrate that the bond interface had been evaluated. An increase in mixed failure after 1 year of water storage was shown in all groups and it could be attributed to degradation in the composite or unprotected collagen fibrils.^2 , 3^ The results of failure mode ( Figure 2 ) shows an equilibrium in the percentage of adhesive failures in both periods of evaluation and this can be interpreted as GM1489 acting on the prevention of dentin-resin bonding degradation.

In general, for polymer-based restorative materials, a high degree of conversion is the first step for the development of clinically-welcomed physicomechanical properties.^29 , 30^ Specifically for adhesive systems, this property is directly related to the efficacy of the bond to dentin.^31^ For example, if an adhesive polymer presents a poor degree of conversion, unreacted monomers in the hybrid layer may leach out over time, thereby creating porosity in its structure that may increase its permeability. This plethora of phenomena favor the hybrid layer degradation, reducing its sealing ability, which might jeopardize the service life of adhesive restorations.^3 , 32^ In the present study, the DC% of experimental adhesives ranged from 98.32% to 99.34%, values that nicely agree with previous studies evaluating commercially available and experimental adhesive systems.^33 - 35^ Most probably, these high values of DC% were influenced by the chemical structure of the monomers used in the adhesive formulations tested here. First, TEGDMA is an aliphatic monomer with high flexibility that increases the adhesive system reactivity.^35^ Second, the “solvent-like” behavior of HEMA may allow a reduction in the adhesive blend viscosity, favoring its reaction with the C=C bonds of long chains even after these are entrapped into the polymer network.^36^ The DC% of SB was statistically lower than those of the experimental adhesives ( Figure 6 ). This result may be explained by the absence of TEGDMA and by the lower concentration of HEMA (5-15%) in SB composition, which could have affected its viscosity and, consequently, its DC%. Different from the experimental adhesive systems, in which the incorporation of GM1489 had no influence on DC%, for SB, the formulations with 5 µM and 10 µM GM1489 presented statistically higher DC٪ ( Figure 6 ). As GM1489 has no polymerizable groups in its structure it was hypothesized that, in these SB formulations, GM1489 could have acted as a spacer, increasing the distance between the monomer and polymer chains during the polymerization reaction. This behavior could have slightly increased the gelation phase of polymerization, thereby allowing more mobility to the terminal C=C bonds to find new polymerizable groups,^37^ positively affecting the DC%. These findings led to the partial acceptance of the third research hypothesis established for the present study.

The second research hypothesis that higher concentrations of GM1489 would be able to reduce the nanoleakage at the interface of adhesive systems was rejected since the nanoleakage results showed no differences among groups for SB and for EXP, in both evaluation times (immediate and after 1 year of storage). Although the nanoleakage values were higher after 1 year of storage, a significant increase in nanoleakage was observed for EXP0 only. However, a trend towards decrease in nanoleakage after 1 year of storage was observed when 10 µM of the inhibitor was used, for both adhesives ( Figure 5 ). These nanoleakage results could be correlated with the DC%, since SB showed higher DC% with 5 µM or 10 µM of inhibitor. High DC% of adhesive systems can contribute to the stability of the resin-dentin bond and lower nanoleakage expression.^29 , 38 , 39^

As was done in the present study, some authors incorporated the MMPs inhibitors into the adhesive system to evaluate their properties, such as µTBS and micropermeability/nanoleakage.^13 - 15 , 17^ All studies showed a trend towards conservation of the hybrid layer in groups with incorporation of the MMP inhibitors. The study of Silva, et al.^14^ (2015) was the first about the use of GM1489 in dentistry, and as was shown in this study, they demonstrated promising results for this inhibitor, which maintained the µTBS stability after 1 year of water storage (similar to chlorhexidine and BB94) and showed a clinically acceptable degree of conversion and lower water sorption than the commercial control without the inhibitor.

## Conclusions

Within the limitations of this study, it could be concluded that 5 µM or 10 µM GM1489 concentrations for experimental adhesive and 5 µM for commercial adhesive should be the choice for the improvement of dentin bonding. Moreover, the DC٪ of adhesive systems and the nanoleakage were not jeopardized by GM1489.
